# Ocular Surface Disease with BAK preserved Travoprost and Polyquaternium 1(Polyquad) preserved Travoprost

**Published:** 2019

**Authors:** Suresh Kumar, Tanu Singh, Parul Ichhpujani, Sanchi Vohra

**Affiliations:** * Department of Ophthalmology, Government Medical College and Hospital, Chandigarh, India

**Keywords:** POAG, OSDI, BAK, polyquad

## Abstract

**Introduction.** The topical medications containing benzalkonium chloride (BAK) as preservative is known to induce corneal toxicity and ocular surface disease (OSD) in glaucoma patients. Newer preservatives like SofZia or polyquaternium-1 (Polyquad) have been developed to replace BAK in many medications. The present study aimed at comparing the OSD in glaucoma patients receiving BAK preserved travoprost versus travoprost with polyquad as preservative and controls not receiving any medications.

**Methods.** This prospective, controlled, observational study was conducted on patients of primary open angle glaucoma (POAG) on medications for more than 6 months. The first group comprised of 40 patients receiving BAK preserved travoprost, the second group included 40 patients receiving polyquad preserved travoprost and 30 of control group not receiving any medical treatment. Ocular Surface Disease Index (OSDI) scores using Ocular Surface Disease Index (OSDI) Questionnaire were assessed and compared in all subjects.

**Results.** The mean OSDI score was 29.09 ± 13.45 in BAK group, 12.4 ± 5.085 in polyquad group and 10.93 ± 7.36 in controls. The mean difference in OSDI scores between BAK and polyquad group 16.63 (p < 0.05) and between the BAK and control group was 18.96 (p < 0.05). The mean difference in OSDI scores between the polyquad and control group was 1.53 (p > 0.05).

The mean IOP in the BAK group was 19.2 ± 3.5 and in polyquad group was 20.1 ± 4.2. The IOP measured at 12 months of treatment was 13.2 ± 2.1 in BAK group and 12.8 ± 3.3 in polyquad group. The IOP measured at baseline and 12 months showed statistically significant difference in both the groups (p <o.oo1, p=o.ooo, respectively).

**Conclusions.** OSDI scores revealed significantly lesser symptoms in polyquad preserved travoprost when compared to BAK preserved travoprost. The OSDI scores in polyquad group were also comparable to the control group. Hence, for long term glaucoma management polyquad containing travoprost should be preferred over the BAK preserved travoprost.

## Introduction

Glaucoma being a chronic progressive disease, intraocular pressure (IOP) remains the most important risk factor that is modifiable to slow the progression of glaucoma [ **1**]. Currently, the first line of management for Primary Open Angle Glaucoma (POAG) patients is IOP reduction by the topical anti-glaucoma medications. Since, most of these drugs are to be administered for a long period of time, these are associated with many local side-effects. Literature suggests that most of these anti-glaucoma medications on prolonged usage are associated with ocular surface disease (OSD) [ **2**- **4**].

Ocular surface disease (OSD) is a group of ocular disorders affecting various components of the ocular surface. 15% of all the patients above the age of 65 years are known to have some form of OSD [ **2**]. One of the studies has reported the prevalence of OSD to be as high as 48-59% Studies indicate a higher prevalence of OSD in glaucoma patients, with one study reporting that about 48-59% in glaucoma patients on topical treatment [ **2**]. OSD is essentially diagnosed clinically by measuring by tear film break up time (TBUT), Schirmer’s test, tear meniscus height (TMH), and observing the staining pattern of the cornea and conjunctiva with the fluorescein dye. However, many of these OSD patients might just complaint of the symptoms inspite of most of the clinical examination tests being in the normal range. Hence, in order to have a more comprehensive assessment of the disease, a 12-item questionnaire called the Ocular Surface Disease Index (OSDI) questionnaire, has been designed [ **5**]. This questionnaire has very well been validated while assessing the OSD in glaucoma patients in particular [ **6**- **9**].

OSD has shown to have a higher prevalence in patients of POAG on anti glaucoma medications. It is not the active ingredient of the drug, but the exposure of various preservatives which contribute to the ocular surface disorders [ **10**]. Benzalkonium Chloride (BAK), one of the most commonly used preservative agent, is a constituent of approximately 70% of the commercially available anti glaucoma drugs [ **11**]. Various clinical studies have shown that BAK induces corneal toxicity, reduces TBUT and aggravates the dry eye disease in glaucoma patients [ **12**, **13**]. Nenciu et al. observed that different anti glaucoma drugs containing BAK as preservative lead to various degrees of conjunctival inflammation and metaplasia [ **14**]. Another study reported loss of goblet cells leading to mucin deficiency type of dry eye disease in patients receiving BAK [ **15**]. BAK due to its disruptive properties might lead to its accumulation in various tissues when used for a long period of time [ **11**].

The safer preservatives that have recently emerged, like polyquaternium-1 (Polyquad®), oxychloro complex, or SofZia® which have shown to have better in various studies [ **16**- **18**]. Various studies done previously have shown significant symptomatic improvement of OSD when BAK preserved latanoprost was switched with BAK free travoprost. [ **7**, **19**, **21**]. Walimbe T et al. reported that switching from BAK containing latanoprost to BAK free latanoprost decreased OSDI scores [ **8**]. All these studies compared BAK preserved and BAK free formulations and showed betterment of the OSDI scores.

Comparing BAK preserved and polyquad preserved anti glaucoma topical therapy with the control group not receiving any medication will give us more information regarding the safety profile of polyquad as a preservative. The present study was formulated to know and compare the OSDI scores in POAG patients receiving BAK preserved prostaglandins, polyquad preserved prostaglandins and controls not receiving any medications.

## Material and methods

A hospital based prospective observational study was done in patients visiting the glaucoma clinic in our center. Study subjects were divided into three groups; the first group comprising of POAG patients receiving BAK preserved travoprost, the second group included POAG patients receiving polyquad-preserved travoprost (Travatan Z, Alcon) and a control group of glaucoma suspects not receiving any medical treatment. 

Patients of POAG with an age of 40 years or more and on single drug prostaglandin therapy (travoprost) with BAK or polyquad for a minimum period of 6 months were enrolled. Patients with pre-existing ocular surface disease prior to institution of medical therapy, corneal abnormalities, prior refractive correction procedures, prior filtration surgery, pregnant or lactating females, and those with other visually significant diseases like cataract, diabetic retinopathy, hypertensive retinopathy, age related macular degeneration (ARMD) were excluded from study. 

POAG was diagnosed based on characteristic optic disc changes with reliable and reproducible glaucomatous visual field loss demonstrated on the Humphrey visual field analyzer (HFA) (Humphrey Instruments, Inc, Zeiss Humphrey, San Leandro, California, USA) with or without raised intraocular pressure and gonioscopically open anterior chamber angle. POAG is a chronic ocular disease process that is progressive, generally bilateral, but can be asymmetric. 

To rule out subjects with pre-existing ocular surface disease, fluorescein staining of corneal surface was done and was graded as (mild staining, less than 10% coverage of corneal surface; moderate, 10%–50% of corneal surface; severe, more than 50% of corneal surface). Presence of moderate to severe staining was considered an exclusion factor.

Patients with the age 40 years or more, refractive errors < 5 D of myopia/ hypermetropia or < 2 D cylinder of astigmatism, non visually significant cataract, normal-appearing optic nerve head, normal visual fields, and not on any anti glaucoma medication, were included as controls. Patients with history of glaucoma or use of anti glaucoma medication previously, ocular hypertension, patients with corneal abnormalities or pre-existing ocular surface diseases, visually significant cataract, diabetic retinopathy and hypertensive retinopathy, were considered as exclusion factor for the control group.

Also, the patients with severe glaucoma getting multiple drugs in the study to avoid misinterpretation due to variable amount of BAK, present in several drugs.

The SITA Fast 24-2 testing algorithm was considered abnormal if the glaucoma hemifield test result was outside normal limits or the pattern standard deviation had a P-value of 0.5%. Optic nerve head and VF findings were then evaluated by glaucoma physicians to rate the probability of glaucoma as definite, probable, possible, or none. 

**Baseline Evaluation**

A careful detailed history of decrease in vision, watering, pain, redness, frequent change of refractive correction, colored halos, headache, and previous history of drug intake, surgery, or ocular trauma was taken. A detailed history of duration since diagnosis of glaucoma, medical therapy including class of anti glaucoma drug, nature of preservative and duration since institution of therapy was taken. Extensive ophthalmologic examination was done for all patients, which included: best corrected visual acuity (measured both with and without correcting glasses by Snellen’s chart at a distance of 6 meters), corneal examination , pupillary reactions, anterior chamber, measurement of intraocular pressure (IOP) with Goldman Applanation Tonometer (GAT), gonioscopy with Zeiss four mirror gonio lens and also complete fundus examination after pupillary dilation with 90D lens.

**Follow Up**

IOP was measured using Goldmann applanation tonometry during on-therapy visits at 6 weeks, 3 months, 6 months, and 12 months, at almost the same time of day (±1 hour). 

**OSDI Questionnaire**

The subjects fulfilling all the inclusion criteria were asked to give an informed written consent and to reply to the questions asked by the investigator from the Ocular Surface Disease Index (OSDI) Questionnaire. OSDI consists of 12 items that assess symptoms, functional limitation, and environmental factors affecting the ocular surface disease.

Each of the 12 items mentioned in the OSDI questionnaire were graded on a scale of 0 to 4. [0=none of the time; 1=some of the time; 2=half the time; 3=most of the time; and 4=all the time]. Using the formula given below, total OSDI score was then calculated and recorded.

OSDI = [(sum of the OSDI score) × 100] / [(total number of questions answered) × 4]

OSDI scores so recorded were then graded on a scale of 0 to 100, where higher score indicated greater disability. Further categorization of the patients was done as those with normal ocular surface (0–12 units), mild ocular surface disease (13–22), moderate (23–32) and severe (33–100) using the OSDI scores.

**Procedure**

Single interviewer assessed all the patients using an orally administered Ocular Surface Disease index Questionnaire. OSDI scores were calculated based on the standard formula as described earlier. Mean and standard deviation was used to analyze descriptive data, while standard deviation was used for quantitative variables. The mean differences along with their 95% CI were presented. Statistical significance was established using independent sample t-test/ ANOVA/Paired t- test/Chi square test. The mean IOP change from baseline at week 12 was analyzed using a paired t-test. P value < 0.05 was considered statistically significant. IBM SPSS version 22 was used for statistical analysis.

## Results

Final analysis included data of 110 people. Among these, 40 people received BAK preserved travoprost, 40 received polyquad preserved travoprost and 30 people were controls and not on any topical treatment. The mean age of patients in the BAK group was 60.88 ± 8.48 years old, polyquad group was 61.25 ± 13.32 years old, and the controls group was 60.42 ± 7.16 years old. No statistically significant difference was seen between the groups (p > 0.05). 

All the study groups had more male patients when compared with the female patients. The proportion of males was 60%, 62.5%, and 60% respectively in BAK, polyquad, and control groups. The differences in the gender composition of participants among the groups was statistically not significant (p > 0.05). The mean duration of receiving treatment in the BAK group was 15.65 ± 9.62 months and 12.83 ± 7.63 months polyquad group. The mean difference (2.83) was statistically insignificant. Baseline mean IOP in the BAK group was (19.2 ± 3.5), (20.1 ± 4.2) in the polyquad group and was (13.3 ± 2.6) in the control group. The IOP measured at 12 months of treatment was 13.2 ± 2.1 in the BAK group and 12.8 ± 3.3 in the polyquad group. The mean difference between the IOP at baseline and at 12 months was statistically significant in both the groups (p value <o.oo1, o.ooo). However, the difference between the mean IOP of two groups at the end of 12 months was statistically insignificant (p value 0.05) ( **Table 1**).

**Table 1:** Demographic profile of subjects

	Controls	Polyquad	BAK
Patients (number)	30	40	40
Age (Years)	60.42 ± 7.16	61.25 ± 13.32	60.88 ± 8.48
Gender (Male/ Female)	18/ 12	25/ 15	24/ 16
Treatment Duration (months)	-	12.83 ± 7.63	15.65 ± 9.62
IOP (mmHg) Baseline	13.3 ± 2.6	20.1 ± 4.2	19.2 ± 3.5
12 months	14 ± 2.4	12.8 ± 3.3	13.2 ± 2.1

**Comparison of mean Ocular Surface Disease score across study groups (N=110**)

The mean Ocular Surface Disease score was 29.09 ± 13.45 in the BAK group, 12.4 ± 5.085 in the polyquad group and 10.93 ± 7.36 in the controls. With these baseline values, the control group had normal ocular surface, the polyquad travoprost had mild ocular surface disease while BAK preserved travoprost group revealed a moderate ocular surface disease. There was statistically significant difference in the OSDI scores BAK and polyquad group (16.63) (p value < 0.05). The mean difference in OSDI scores between the BAK and control group (18.96) was statistically significant (p value < 0.05). Higher OSDI scores in the BAK group than in the polyquad group indicated more severe OSD in the BAK group. The mean difference in OSDI scores between the Polyquad and the control group (1.53) was statistically insignificant (p > 0.05). Similar OSDI scores in the polyquad and the control group indicated similar OSD in both these groups ( **Table 2**).

**Table 2:** Comparison of mean OSDI score across study groups (N=110)

Preservative	Ocular Surface Disease score Mean ± Std. Dev	Mean difference	95% Confidence Interval for Mean		P value
			Lower Bound	Upper Bound	
Control (N=30)	10.93 ± 7.36	Baseline			
Polyquad (N=40)	12.45 ± 5.08	1.53	-4.04	7.10	1.00
BAK (N=40)	29.09 ± 13.45	18.96	12.59	23.73	<0.01
Polyquad (N=40)	12.45 ± 5.08	Baseline			
BAK (N=40)	29.09 ± 13.45	16.63	11.47	21.79	<0.01

There was no significant difference in the occurrence of conjunctival hyperaemia between the study drugs and BAK-free travoprost was well tolerated.

Further analysis of the OSDI questionnaire focusing on the problems relating to symptoms (question 1 – question 5), functional limitation (question 6 - question 9) and environmental factors affecting the ocular surface, was done in the polyquad travoprost and BAK preserved travoprost group ( **Table 3**).

**Table 3:** Comparison of question specific analysis of OSDI in the Polyquad travoprost and BAK-preserved travoprost group

OSDI Questionnaire	Polyquad group	BAK group
Q1 - Q5 (Symptoms)	6.3 +/ - 2.62	14.8 +/ - 6.57
Q6 - Q9 (Functional limitation)	2.2 +/ - 0.73	5.6 +/ - 2.76
Q10 - Q12 (environmental factors affecting the ocular surface)	3.9 +/ - 1.73	8.69 +/ - 4.12

The analysis revealed that in either group the symptoms got worse in the adverse environmental conditions like windy conditions, in air-conditioned rooms and in places with low humidity. Also, the subset of the elderly patients who had lesser outdoor exposure seemed to be less symptomatic and thereby had a lower OSDI score.

## Discussion

Preservatives are unavoidable, being required to maintain sterility of topical medications. All preservatives are known to affect ocular surface structure as well as function. However, BAK is notorious for its effects on ocular surface tissues, due to its detergent like action. Polyquad, a derivative of BAK, is much less toxic to ocular tear film because of its large molecular size and less hydrophilic property [ **16**, **17**]. Other preservatives like SofZia, an ionic buffer and oxychloro complex breaks down into nontoxic substances in tear film [ **16**, **18**]. However, all preservatives can aggravate or cause OSD in glaucoma patients.

In our observational, cross sectional prospective study including 40 patients getting BAK preserved travoprost drops, 40 patients on polyquad preserved travoprost and 30 controls not getting any treatment, OSDI questionnaire confirmed statistically significantly higher OSDI scores in patients receiving BAK preserved travoprost in comparison to patients receiving polyquad preserved travoprost (p < 0.05). OSDI scores were seen to be comparable between the polyquad preserved travoprost and the control group not receiving any medications (p > 0.05).

Mean age of the patients in the BAK, polyquad and control groups were comparable (p > 0.05). The proportion of males was 60%, 62.5%, and 60% respectively in BAK, polyquad, and control groups, which was also comparable (p value 0.97). The mean age and male preponderance as seen in our study were similar to that seen by Skalicky et al. [ **9**].

Our study documented statistically significant decrease in IOP in both the polyquad preserved group and the BAK preserved group, with a mean IOP reduction of 7.2 mmHg and 6 mmHg from baseline respectively (p <o.oo1, p=o.ooo, respectively). IOP reduction in similar range was observed by by Gandolfi et al. [ **20**], where the mean IOP reduction of 7.6 – 8.7 mmHg was seen in the travoprost BAK-free group and 7.7 – 9.2 mmHg in the travoprost BAK group.

**OSDI Scores: BAK versus polyquad preserved travoprost**

The mean OSDI scores were statistically significantly higher in the BAK group as compared to the polyquad group (p < 0.05) in our study, implying that ocular surface was more affected in the BAK group than the polyquad group. Katz et al. and Lopes et al in their separate studies also validated this fact that OSDI scores were significantly higher in the BAK preserved group as compared to the BAK free group [ **7**, **21**]. One prospective study found that the mean OSDI scores decreased significantly (p < 0.001) after switching the BAK preserved latanoprost to SofZia preserved travoprost [ **18**]. Switching BAK preserved latanoprost to BAK free latanoprost led to significant decrease in the OSDI scores at the end of 56 days in an Indian study (p < 0.0001) [ **8**]. Looking into the literature and comparing it with our results, shifting of patients from the BAK containing topical prostaglandin analogues to polyquad containing prostaglandin analogues seems like the most logical step to improve the ocular surface status of the patients.

**OSDI Scores: BAK preserved versus controls**

While comparing the controls with the BAK preserved group, OSDI scores were significantly worse in the BAK preserved group (p < 0.05). Skalicky et al. not just observed worse OSDI scores in the BAK group but also found that daily instillation of BAK preserved drops with a frequency of more than 3 times was predictive of higher OSDI (Odds ratio of 2.47) [ **9**]. Saade et al. also found to have higher OSDI scores were found to be higher in the BAK preserved medication group than in controls not receiving any medication (18.97 ± 9.5 versus 6.25 ± 5.7) [ **22**]. Pérez-Bartolomé F reported significantly higher OSDI scores in the preserved medication group compared to controls (p < 0.001) [ **23**]. The findings of significant higher OSDI scores in BAK preserved versus control groups in the present study were consistent with the other published articles in literature. Therefore, it can be concluded that BAK leads to significantly higher OSD in glaucoma patients as compared to controls not receiving any topical medication.

**OSDI Scores: Polyquad preserved versus controls**

The OSDI scores were not found to be statistically significantly different in the polyquad preserved medications and the control group (p > 0.05), indicating that the severity of OSD in the polyquad group was not significantly different from the controls. The study by Skalicky et al. divided glaucoma patients into mild, moderate, and severe groups with all patients receiving different doses of BAK [ **9**]. It also included a control group, which was compared with the BAK group showing a significant difference. However, the study did not compare the BAK free group with the controls, as no BAK free group was included.

In a recent study by Rolle T et al., the OSDI scores were found to be significantly higher in preservative free tafluprost group and the preservative free timolol group than in controls (p = 0.0000) [ **24**]. However, unlike this study, we found the OSDI scores in the polyquad group not to be significantly different from the control group. The minimum duration of treatment in their study was 36 months, whereas in our study the IOP lowering effect was measured at 12 months of treatment. The higher OSDI scores seen with this study can be due to long-term side effect of tafluprost or timolol treatment.

The Indian data comparing polyquad-preserved medications with controls not receiving any medications is scanty. Our study included 3 groups and compared the BAK group and the polyquad group with controls not receiving any treatment and we observed that the polyquad containing medications did not significantly increase the OSDI scores in comparison to controls 12 months after the start of treatment. Hence, the polyquad preserved topical prostaglandins could be used safely without a significant increase of OSD in glaucoma patients. This essentially meant that shifting the BAK preserved prostaglandins to polyquad preserved prostaglandin analogue improved long-term tolerance in these patients. 

Preservatives like BAK can increase the efficacy of the drug due to the enhancement of the penetrative property of the basic molecule by its lipophilic action. Polyquad being less lipophilic might decrease the penetration of the active ingredient and thereby decreasing its absorption and efficacy [ **16**]. However, both BAK preserved and polyquad preserved anti glaucoma drugs have shown similar decrease in the IOP in a study by Lopes JF et al. [ **21**]. Similarly, Walimbe T and co-authors observed that substituting BAK free latanoprost from BAK preserved latanoprost had no effect on IOP lowering efficacy after 56 days of treatment [ **8**]. Another study found both BAK containing and BAK free drugs to be equally efficacious in reducing IOP in POAG patients and Ocular Hypertension [ **10**]. In our study also we observed similar IOP reduction in polyquad-preserved medication and BAK preserved anti glaucoma medication (p value 0.05) at the end of 12 months. In our study, the mean treatment duration was 12 months, which was longer than in most of other studies having the mean duration of treatment of around 3 months. Thereby validating the fact that, changing to polyquad as preservatives is not likely to affect the long-term efficacy of the anti glaucoma medication.

Few limitations in the present study were that all the patients received monotherapy with different generic brands of travoprost. Some selection bias might have been induced since the cases and controls were selected from subspecialty glaucoma practices. The strong point in our study was the good sample size in all the groups. OSD was assessed using OSDI questionnaire, which was a universally accepted tool for assessing these patients. A single observer interviewed all the patients with questionnaire, which has eliminated the perceived bias in the study protocol. We also included a control group without medications, which was compared with polyquad group to get better information.

## Conclusion

Polyquad preserved antiglaucoma medication is associated with significantly lesser symptoms of ocular surface disease than the BAK preserved drugs as seen with the use of OSDI questionnaire. Also, the OSDI scores in the polyquad group were almost similar to those in the control group implying that the polyquad does not worsen the ocular surface disease. The IOP lowering efficacy of polyquad-preserved prostaglandins was found to be similar to BAK preserved prostaglandins at the end of 12 months of follow up. Therefore, polyquad preserved prostaglandin must be safe and effective in the management of POAG patients.

**Conflicts of Interest**

Nil. 

**Source of Funding**

Nil. 
